# Helicobacter Pylori Variants with ABC-Type Tyrosine Phosphorylation Motif in Gastric Biopsies of Ghanaian Patients

**DOI:** 10.1155/2021/6616059

**Published:** 2021-03-30

**Authors:** Emmanuel A. Tagoe, Gordon A. Awandare, Osbourne Quaye, Richard H. Asmah, Timothy N. Archampong, Mahasin A. Osman, Charles A. Brown

**Affiliations:** ^1^West African Centre for Cell Biology of Infectious Pathogens (WACCBIP)/Department of Biochemistry, Cell and Molecular Biology, University of Ghana, Legon, Accra, Ghana; ^2^Department of Medical Laboratory Sciences, University of Ghana, Korle Bu, Accra, Ghana; ^3^Department of Biomedical Sciences, School of Basic and Biomedical Sciences, University of Allied Health Sciences, Ho, Ghana; ^4^Department of Medicine, University of Ghana Medical School, University of Ghana, Korle Bu, Accra, Ghana; ^5^Departments of Medicine, College of Medicine and Life Sciences, University of Toledo, OH 34614, USA

## Abstract

**Background:**

*Helicobacter pylori* pathogenicity and disease severity are determined by the tyrosine phosphorylation motifs of CagA protein. This study is aimed at detecting the presence of *H*. *pylori* and identifying the CagA tyrosine phosphorylation motifs in Ghanaian patients. *Material and Methods*. A total of 94 archival genomic DNA samples from gastric biopsies were used for the study, and *H*. *pylori* was detected by amplifying the 16S rRNA gene. The 3′-end variable region of the cagA gene was amplified, and the entire 3′-end was sequenced and translated into amino acids.

**Results:**

*H*. *pylori* was detected in 53.2% (50/94) of the samples, and all the detected bacteria harboured the *cagA* gene. Two variants of the bacteria were identified based on the size of the amplified *cagA* gene: 207 bp and 285 bp. The 207 bp and 285 bp variants accounted for 74% and 22%, respectively, and 4% showed both fragments. Translated amino acid sequence of the *cagA* gene showed EPIYA-A, EPIYA-B, and EPIYA-C (ABC type) motifs, indicating the Western variant. The CagA protein C-terminal showed insertion of amino acids in the sequence flanking the EPIYA-A motif at the N-terminal and a complete deletion of the EPIYA-CC and EPIYA-CCC motifs together with the flanking sequences.

**Conclusions:**

*H*. *pylori* identified were Western variant (ABC type) with unique amino acid insertions, suggesting unique variants in Ghanaian patients. Further investigation is however required to understand the role of the molecular diversity of the variant in gastric disease outcome.

## 1. Introduction

*Helicobacter pylori* (*H*. *pylori*) is a major etiological agent of human gastric disease, and almost half of the world's population is infected with the pathogen [1]. Infection occurs in early childhood, and the bacteria transmission has been associated with socioeconomic factors [2]. *H*. *pylori* variants are diverse across populations [3], and the diversity has been attributed to polymorphism of virulent genes encoded by a 40 kb *cag*-pathogenic island (*cag*-PAI) [4]. The cytotoxin-associated A (c*agA*) and vacuolating cytotoxin (*vacA*) genes are among the well-studied *H*. *pylori* genes of the *cag*-PAI that account for the pathogenicity of the bacteria [5]. Though, almost 70% of *H*. *pylori* variants globally contain the *cagA* gene, the distribution of the *cagA*-positive variant varies geographically [6]. A prevalence of 90-95% *cagA-*positive variants has been reported in East Asia as against 60% in Western countries [6]. The *cagA* gene encodes a 120-145 kDa oncogenic CagA protein which is translocated into the host cell via a type IV secretion system [7]. The oncogenic effect of the protein in the cell largely depends on a highly polymorphic C-terminal region [8, 9]. Within the CagA C-terminal variable region are repeated Glu-Pro-Ile-Tyr-Ala (EPIYA) motifs. The motifs were assigned EPIYA-A, EPIYA-B, EPIYA-C, and EPIYA-D, and the assignments depend on the amino acid sequences that flank the motifs [6]. The EPIYA motifs, together with the flanking amino acid sequences, delineate *H*. *pylori* as either Western or East Asian variant [10]. The Western strain contains conserved EPIYA-A and EPIYA-B motifs in addition to a single or repeated EPIYA-C, while the East Asia strain contains EPIYA-D and the conserved EPIYA-A and EPIYA-B motifs [11].

The Western strain, which habours a greater number of repeated EPIYA-C motifs, has been shown to be more pathogenic than variants with less number of the motifs, but not as pathogenic as the East Asian strain with the EPIYA-D motifs [12]. The EPIYAs are the sites for CagA phosphorylation in host cells, and the phosphorylated or nonphosphorylated protein has been implicated in epithelial cells' morphological changes and gastric disorders [11]. Evidence suggests that phosphorylation of the EPIYA sites is preferential; EPIYA-A and EPIYA-C phosphorylation occurs in the Western strain, and EPIYA-B and EPIYA-D occurs in the East Asian strain [13].

As a mechanism underlying CagA-associated pathogenesis, translocated and phosphorylated CagA tethers at the plasma membrane and recruits SH2 domain-containing protein tyrosine phosphatase-2 (SHP2) from the cytoplasm to the plasma membrane [14]. The number of EPIYA motifs potentiates the recruitment of SHP2 and enhances CagA-SHP2 interaction, leading to cell-cell contact disruption, cytoskeletal rearrangement, and cell elongation [15]. The EPIYA-D-containing CagA more strongly interacts with SHP2 than the protein with the EPIYA-C motif, and the interaction increases SHP2 phosphatase activity in the East Asia strain compared to the Western strain [16]. Interaction of CagA with SHP2 via the EPIYA motifs has been shown to predispose individuals infected with *H*. *pylori* to precancerous lesion and gastric cancer [17]. Strain diversity of the bacteria, based on the CagA variants, has therefore been implicated as an important tool in predicting risk of developing gastric dysplasia and also serving as a prognostic marker [18].

In a recent study among Ghanaian patients diagnosed with gastric dysplasia, *cagA* gene was more likely to be detected in patients with duodenal ulcer [19]. However, no study has characterized *H*. *pylori cagA* gene based on the amino acid sequences of the C-terminal to determine bacteria diversity in Ghana. The current study is aimed at investigating the diversity of cagA-positive *H*. *pylori* variant in Ghana by amplifying the 3′-end variable region using multiplex PCR and performing amino acid sequence alignment to determine the predominant EPIYA motifs of the variant in the population.

## 2. Material and Methods

Archival frozen genomic DNA samples obtained from patients diagnosed with gastric disease at Korle Bu Teaching Hospital in the southern part of Ghana were used in the current study. A total of 94 genomic DNA samples were retrieved, and PCR was used to detect the presence of the bacteria by amplifying the *16S rRNA* gene. The samples found to contain *H*. *pylori* DNA were selected for further PCR analysis to identify *H*. *pylori cagA* gene 3′-end variable region. DNA extracted from cagA-positive *H*. *pylori* (ATCC 435403) strain, using a bacterial genomic DNA purification kit (Wizard Genomic DNA Purification Kit, Promega, USA), was used as control. The control DNA concentration and purity were determined using NanoDrop 2000/2000c (Thermo Fisher Scientific, USA) spectrophotometer and stored at -20°C until ready to use.

### 2.1. Amplification of *H*. *pylori 16S rRNA* and *cagA* Gene 3′-End Variable Region

Detection of *H*. *pylori* 16S rRNA gene in the genomic DNA samples was by PCR using a set of primers (Table 1) and ready-to-use OneTaq Quick-Load 2X Master Mix (BioLabs Inc., England) by following the manufacture's reaction conditions. Each 25 *μ*L PCR reaction mixture had the following components and final concentrations: 1x reaction buffer, 0.5 *μ*M each of the forward and reverse primers, and 0.1 *μ*g/*μ*L genomic DNA. Nuclease-free water was added to obtain the reaction volume. The reaction mixture was subjected to an initial denaturation at 94°C for 30 seconds, 30 cycles of denaturation at 94°C for 30 seconds, annealing at 68°C for 1 minute, and extension at 68°C for 1 minute. Final extension was performed at 68°C for 5 minutes.

The *cagA* gene 3′-end variable region was amplified from genomic DNA samples found to contain *H*. *pylori* DNA using multiplex PCR. Each 50 *μ*L PCR reaction mixture had the following components and final concentrations: 1x Go Green Go *Taq* pol reaction buffer, 1.25 units Go Taq G2 DNA pol, 0.25 *μ*M of each dNTP, 0.5 *μ*M of each primer, and 0.2 *μ*g/*μ*L genomic DNA sample. Nuclease-free water was added to make up the reaction volume. The PCR machine was programmed at the following cycling conditions for the amplification of the targeted gene: initial denaturation at 94°C for 1 minute, 35 cycles of denaturation at 94°C for 55 seconds, 53.1°C annealing for 1 minute, and 72°C extension for 1 minute and 30 seconds. Final extension was at 72°C for 5 minutes. The sets of primers used for *cagA* gene characterization and expected amplification product sizes are shown in Table 1.

In both PCR setups, the extracted control DNA sample and PCR reaction mixture without DNA material were used as positive and negative controls, respectively. The PCR amplifications were carried out using the SensQuest Labcycler (Hannah Vogt Str, Germany), and the 16S rRNA and *cagA* gene 3′-end variable region products were separated on 1.5% and 2.5%, respectively, agarose gels with ethidium bromide in tris acetate-EDTA (TAE) buffer. A molecular weight ladder of 100 bp was used to determine the size of the bands.

### 2.2. Selection of Genomic DNA Samples for *cagA* Gene 3′-End Variable Region Sequencing

The DNA samples for commercial sequencing were selected based on the fragment sizes of the PCR amplified *cagA gene* 3′-end variable region that has been separated on 2.5% agarose gel. The representative samples for sequencing were a single band with molecular weight of either 207 bp or 285 bp and a double band with molecular weights of 207 bp and 285 bp. Sample tubes containing the selected genomic DNA samples were clearly labelled and shipped for sequencing with instructions. The primer set that was used for the *cagA* 3′-end region amplification and sequencing is previously described [20].

### 2.3. Sequence Alignment

The translated CagA protein amino acid sequences in FASTA format were trimmed and aligned with similar sequences obtained from NCBI GenBank database using the European Molecular Biology Laboratory-European Bioinformatics Institute (EMBL-EBI) platform.

## 3. Results

### 3.1. Identification of *H. pylori by 16S rRNA* Amplification Using Conventional PCR

**Figure 1:**
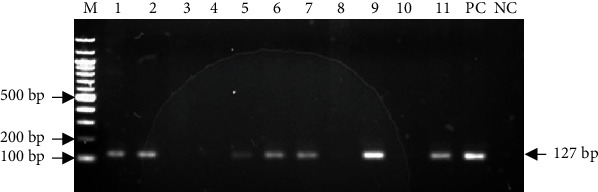
Detection of H. pylori *16S RNA* gene in Ghanaian isolates. A representative of ethidium bromide-stained 1.5% agarose gel electropherogram. Lane M = 100 bp molecular.

The 16S rRNA gene of *H. pylori* was amplified in 50 (53.2%) out of the 94 samples. *H*. *pylori* 16S rRNA gene fragments and predicted size are shown in Figure 1.

### 3.2. Amplification and Percentage Distribution of *H. pylori cagA* Gene 3′-End Variable Region

**Figure 2:**
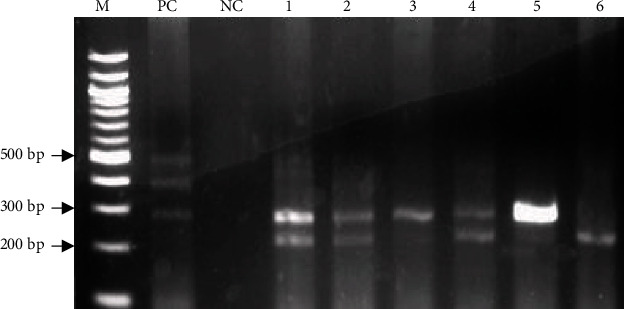
Detection of *cagA* gene 3′-end variable sequence in Ghanaian isolates. Representative ethidium bromide-stained 2.5% agarose gel electropherogram of multiplex PCR for the identification of the *H*. *pylori cagA* 3′ variable end sequence. Lane M = 100 bp molecular weight ladder; lanes 3 and 5: amplification of a single variable sequence of *cagA* gene of *H. pylori* strain with band size of 285 bp, and lane 6 = 207 bp. Lanes 1, 2, and 4: amplification of double variable sequences of *cagA* gene with band sizes 207 bp and 285 bp; lane PC: positive control DNA of *H*. *pylori* strain (ATCC 43504) showing three *cagA* gene 3′-end variable sequences (ABCCC); NC: negative control.

All the 50 samples that were positive for *16S rRNA* gene showed the presence of *cagA gene* 3′-end variable region. Based on the sizes of the amplified fragments (Figure 2), two different *H*. *pylori* variants were detected in the samples: a 207 bp and 285 bp.

**Figure 3:**
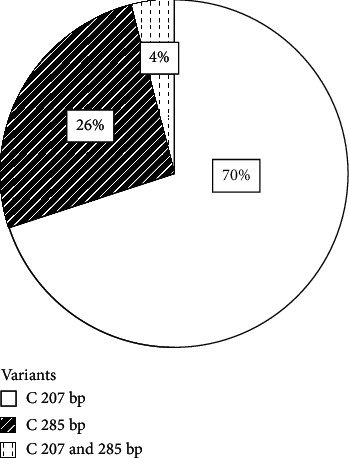
Percentage distribution of *H*. *pylori cagA* gene 3′-end variable region in the study samples.

Percentage distribution of the *cagA* gene 3′-end variable sequences in the samples is shown in Figure 3. Bacteria variants that harboured variable *cagA* gene 3′-end variable sequence of band size 207 bp, indicating a single infection (70%), were the most predominant compared to the mixed infection and the 285 bp single infection type.

### 3.3. Alignment of Amino Acid Sequences of CagA Protein C-Terminal with Reference Sequences

**Figure 4:**
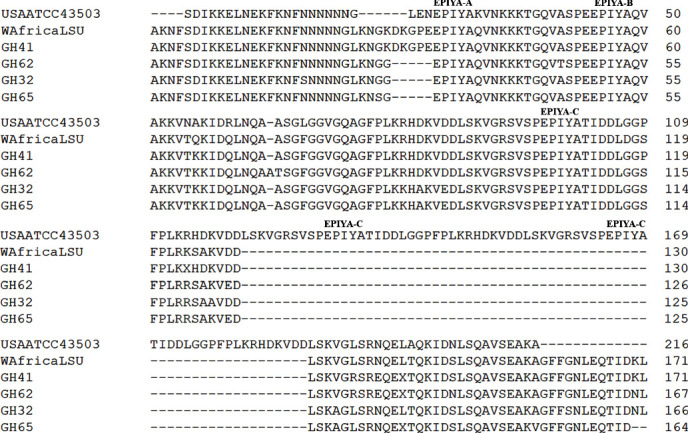
Variation of *H*. *pylori* CagA protein C-terminal in the Ghanaian patients. Alignment of amino acid sequences of the C-terminal in representative samples with reference sequences. The amino acid sequences and EPIYA motifs are presented in segments, and the numbers indicate the cumulative amino acid residues per sequence. GH: study variant from Ghana. Reference sequences are USA43504 (BAB20926.1) and WAfricaLSU (CVB36400.1). Reference sequences were retrieved from NCBI GenBank database.

Alignment of amino acid sequences of cagA-positive *H*. *pylori* variants from the study samples with reference sequences from NCBI GenBank database is presented in Figure 4. The reference (USAATCC43503) sequence showed the conserved EPIYA-A and EPIYA-B and three variable EPIYA-C motifs (ABCCC type), while the WAfricaLSU strain EPIYA motifs were similar to the variants identified in this study. The sequences from the current study were obtained from a 207 bp variant from the samples GH32 and GH62 with amino acids glycine, leucine, lysine, arginine, glycine, and glycine (GLKNGG) insertions and GH65 with a serine substitution (GLKNSG), at the N-terminal flanking sequence of EPIYA-A motif.

The 285 bp variant (GHA41) showed amino acids glycine, leucine, lysine, arginine, glycine lysine, aspartic acid, lysine, glycine, proline, and glutamic acid (GLKNGKDKGPE) insertions with a sequence pattern similar to the WAfricaLSU reference strain. The amino acid sequences of all the amplified fragments showed conserved EPIYA-A and EPIYA-B motifs as well as a single EPIYA-C motif which occurred at the first variable region of the CagA protein.

## 4. Discussion

*H*. *pylori* is regarded as one of the most commonly diverse microorganisms ever studied [21, 22]. Possible explanations for the strain diversity include the ability of the organism to excise short damaged nucleotide sequences using type III endonucleases and explore nucleotide excision repair pathways for bulky lesion repair and importation of exogenous DNA through the cytoplasmic membrane channel during DNA repairs [21, 22].

Identification of the virulence factors and gene differences of *H*. *pylori* in a population is crucial since bacterial pathogenicity and diseases disparity largely depend on these factors [23]. The virulence factors, together with diet and host-pathogen response mechanism, play important roles in gastric disease outcome. The CagA protein is the most studied virulence factor encoded by a 40 kb cag-pathogenic island (cag-PAI), and the island is responsible for several bacterial virulence factors including type IV secreting system (T4SS) required for translocation of the CagA protein into the host cell [24]. cagA-positive *H*. *pylori* strain is associated with gastric disease compared to cagA-negative strain, and disease aggressiveness in patients is linked to genetic variability of the CagA protein [12].

The current study presents, for the first time, information on the CagA C-terminal variable region of *H*. *pylori* variants obtained from archival genomic DNA that was previously extracted from stomach biopsies of Ghanaian patients with gastric disease. This study showed a lower prevalence of cagA*-*positive strain compared to a previous study from the same population which showed a prevalence of 74.8% [19]. The relatively low detection of bacterial DNA reported in the current study may be due to the over 24-month storage of the genomic DNA that was used. An inverse relationship between amount of DNA and storage time has been reported in a study in which microbial DNA depreciated at a rate of 6.6% per 10 days when samples were stored at -20°C [25].

Our study showed two pathogenic *H*. *pylori* variants. The difference in the two variants was the different fragment size of the *cagA* 3′-end variable region; the smaller fragment variant was more predominant and showed a high prevalence of single infection. The two variants however share similar sequences at the CagA C-terminal variable and had EPIYA-A, EPIYA-B, and EPIYA-C motifs with amino acid insertion at the N-terminal sequence flanking the EPIYA-A motif. Even though other studies have shown similar variations in the size of the *cagA* gene 3^″^-end variable region [10, 20], the variants from this study had a complete deletion of EPIYA-CC and EPIYA-CCC and the flanking sequences.

This study reports 100% Western-type *H*. *pylori* variants (ABC type) in the gastric biopsies that were used. A high prevalence of CagA ABC type monoinfection has been reported in patients diagnosed with gastric disease [26], and variants with more than one EPIYA-C motifs were associated with disease severity [27]. Patients diagnosed with gastric ulcer and cancer have been found to harbour *H*. *pylori* strains with EPIYA-ABCCC and ABCC motifs, respectively [10, 28], and the number of motifs correlated with inflamed cells [29]. On the contrary, a study observed that a single EPIYA-C motif phosphorylation was sufficient for cellular perturbation [30], and proinflammatory response to *H*. *pylori* infection was independent of the number of CagA EPIYA-C motifs that was phosphorylated [31].

The carcinogenic potential of CagA protein is determined by the dynamics of the tyrosine phosphorylation of EPIYA-C repeats [29, 32], and the intracellular phosphorylation exhibits preferential targeting of the EPIYA sites [13]. Phosphorylated EPIYA motifs result in the hummingbird phenotypic appearance of gastric cell lines, hall mark of cancers [33]. The mechanism of pathogenic *H*. *pylori*-induced cell morphological changes is through plasma membrane tethering of phosphorylated CagA and recruitment of SH2 domain-containing protein tyrosine phosphatase-2 (SHP2) from the cytoplasm to the plasma membrane [14, 30]. The number of EPIYA motifs has been reported to potentiate SHP2 recruitment [15]. Plasma membrane tethering and phosphorylation of CagA are prerequisite for CagA-SHP2 interaction, and both processes require the EPIYA motifs [14]. The CagA-SHP2 interaction disrupts cell signalling leading to malignant transformation of mammalian cells via focal adhesion kinase (FAK) dephosphorylation [34–36]. The molecular episode depolarizes gastric epithelial cells, disrupting gastric secretions and promoting chronic *H*. *pylori* colonization in the stomach [37].

The EPIYA phosphorylation differences were reported to account for the gastric pathophysiological actions of *H*. *pylori* strains [38]. Even though the importance of the amino acid substitution or sequence insertion in the study variants is not known, specific amino acid sequences within the CagA 3′-end variable region have been reported to play a role in CagA protein translocation [39]. The current detected EPIYA variants and amino acid sequence differences, together, may explain the increased infection in the developing countries but low prevalence of gastric cancer cases.

## 5. Conclusion

In conclusion, the Western strain (ABC type) of *H*. *pylori* was the most predominant strain in the study population, and the two *H*. *pylori* variants detected differed by the size of the amplified fragment. Both variants showed amino acid substitution or sequence insertion at the N-terminal sequence flanking the EPIYA-A and deletion of the second and third EPIYA-C motifs together with the flanking amino acid sequences. A larger sample size is required to confirm the preliminary data in the Ghanaian populace and to investigate further the clinical importance of the insertions and the presence of the single EPIYA-C motif in the CagA C-terminal variable region in gastric disease outcome.

## Figures and Tables

**Table 1:** Sets of primers for *H*. *pylori* detection and characterization.

Target gene	Primer name	Primer sequence (5′-3′)	Tm (°C)	Expected product size (bp)
*H. pylori 16S rRNA*	F^a^	TCCAACAACTAGCATCCATC	52.2	127
	R^a^	AGGAATACTCATTGCGAAGG	52.2	
Western type	WF	ATGATCTCGGCGGACGACCTTT	60.7	207, 285, 387, and 489
	WR	TGCGTGTGTGGCTGTTAGTAG	57.3	
East Asia type	EF	GCATCAGCAGGTAAAGGAGTG	58.8	557

*H*. *pylori 16S rRNA* primers (this study): F^a^: forward primer; R^a^: reverse primer. Western type: WF: forward primer; WR: reverse primer. EF: East Asia type forward primer [20].

## Data Availability

All data generated or analysed during this study are included in this published article.
